# Crystal structure and MD simulation of mouse EndoV reveal wedge motif plasticity in this inosine-specific endonuclease

**DOI:** 10.1038/srep24979

**Published:** 2016-04-25

**Authors:** Meh Sameen Nawaz, Erik Sebastian Vik, Mia Elise Ronander, Anne Marthe Solvoll, Pernille Blicher, Magnar Bjørås, Ingrun Alseth, Bjørn Dalhus

**Affiliations:** 1 Department of Microbiology, Oslo University Hospital HF, Rikshospitalet and University of Oslo, NO-0424 Oslo, Norway; 2 Department of Medical Biochemistry, Institute for Clinical Medicine, University of Oslo, NO-0424 Oslo, Norway

## Abstract

Endonuclease V (EndoV) is an enzyme with specificity for deaminated adenosine (inosine) in nucleic acids. EndoV from *Escherichia coli* (EcEndoV) acts both on inosines in DNA and RNA, whereas the human homolog cleaves only at inosines in RNA. Inosines in DNA are mutagenic and the role of EndoV in DNA repair is well established. In contrast, the biological function of EndoV in RNA processing is largely unexplored. Here we have characterized a second mammalian EndoV homolog, mouse EndoV (mEndoV), and show that mEndoV shares the same RNA selectivity as human EndoV (hEndoV). Mouse EndoV cleaves the same inosine-containing substrates as hEndoV, but with reduced efficiencies. The crystal structure of mEndoV reveals a conformation different from the hEndoV and prokaryotic EndoV structures, particularly for the conserved tyrosine in the wedge motif, suggesting that this strand separating element has some flexibility. Molecular dynamics simulations of mouse and human EndoV reveal alternative conformations for the invariant tyrosine. The configuration of the active site, on the other hand, is very similar between the prokaryotic and mammalian versions of EndoV.

The exocyclic amine group of adenosine (A) can be hydrolyzed, resulting in formation of inosine (I). This process occurs spontaneously in cells and is induced by certain types of stress, for example from nitrosative agents formed as a response to inflammation or infection, or from exposure from the environment^1^. Inosine is read as guanosine (G) and consequently cytosine (C) is incorporated opposite inosine by the DNA polymerases during replication^2^. The A-to-I conversion is thus mutagenic. Removal of inosines from genomic DNA is dealt with by the base excision repair pathway^3^,^4^,^5^ as well as endonuclease V (EndoV), which is considered the major player^6^,^7^. In *Escherichia coli* and some other bacteria, EndoV initiates repair by cleavage of the second phosphodiester bond 3′ to inosine in an Mg^2+^ dependent reaction^8^,^9^,^10^,^11^. In contrast to DNA glycosylases, EndoV does not remove the deaminated nucleotide. After cleavage, EndoV stays tightly bound to the incised product^12^,^13^ and additional, yet unknown, proteins are recruited to complete the repair^14^. Weak activity for inosines in DNA has been reported for mouse (m)EndoV^15^ and human (h)EndoV^16^, however some controversy exists^17^.

Whereas inosine in DNA is considered as damage, inosine in RNA is introduced by specific enzymes in a highly regulated manner to increase transcriptomic diversity. The adenosine deaminases acting on RNA (ADARs) enzymes catalyze this A-to-I editing on mRNA and non-coding (nc)RNA including long ncRNA, micro (mi)RNA and small interfering (si)RNA. A-to-I editing is abundant in higher eukaryotes and edited sites amount to more than a hundred million and are spread over the majority of human genes^18^. Defect editing is linked to various human diseases including neurological disorders, infections and cancer^19^. Also some tRNAs undergo A-to-I editing and here the reaction is catalyzed by enzymes homologous to ADARs, namely the adenosine deaminases acting on tRNA (ADATs). This editing is essential for protein synthesis^20^. Unexpectedly, recently it was shown that both human and *E. coli* (Ec)EndoV could incise RNA substrates containing inosines. RNA cleavage was catalyzed with comparable efficiencies for the two enzymes, comparable to that of DNA for EcEndoV, suggesting that RNA is the preferred substrate *in vivo* at least for hEndoV^17^,^21^. The biological significance of RNA incision at inosines by EndoV is yet not known^22^,^23^.

Some links between EndoV and RNA metabolism was already known from analysis of known 3D structures. For example, the crystal structure of EndoV from *Termotoga maritima* (TmEndoV) in complex with inosine-containing DNA has been solved^12^. This structure reveals that TmEndoV contains an “RNase H-like motif” resembling that in *E. coli* RNase H and the PIWI domain of *Pyrococcus furiosus* Argonaute, both being well characterised RNases^12^. As no robust DNA repair activity has been found for the mouse EndoV homolog, we speculated whether mEndoV also is an RNase with preference for inosines in RNA. Indeed mEndoV cleaved inosine-containing RNA, albeit less efficient than hEndoV. The crystal structure of mEndoV was solved and when compared to the structure of hEndoV, we observe a conformation of the strand-separating wedge, and a concurrent wide DNA/RNA binding cleft, different from all previous EndoV structures (prokaryotic and human). The new structure suggests that this conserved element is flexible and may switch between unproductive and productive conformations for RNA cleavage.

## Results

### Biochemical properties of mEndoV

**Figure 1:**
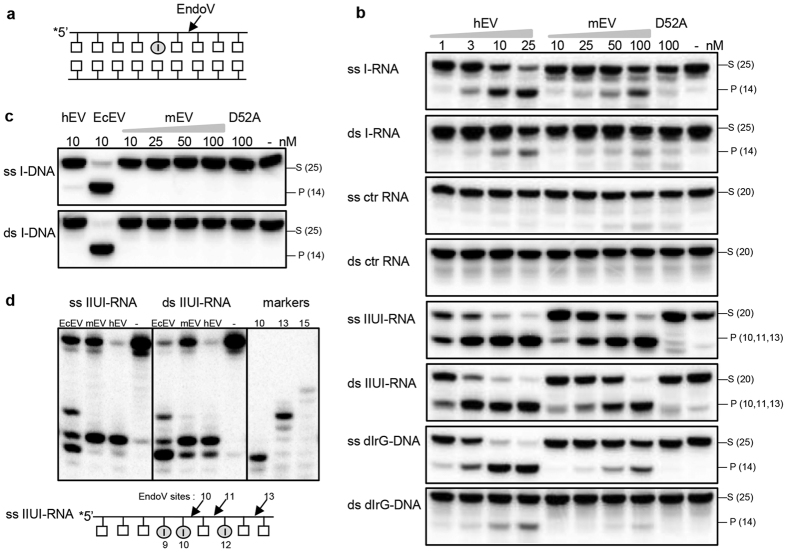
Processing of inosine by EndoV enzymes. (**a**) Schematic illustration of EndoV cleavage of the second phosphodiester bond 3′ to an inosine residue. (**b**) Increasing amounts of human EndoV (hEV;1–25 nM) and mouse EndoV (mEV; 10–100 nM) were incubated with 1 nM RNA or DNA substrates: ss/ds I-RNA, ss/ds ctr-RNA, ss/ds IIUI-RNA, ss/ds dIrG-DNA and (**c**) ss/ds I-DNA at 37 °C for 30 minutes using standard reaction buffer. 100 nM of the site specific mEndoV mutant D52A was included in all assays. Cleavage products were analyzed by 20% denaturing PAGE and visualized by phosphorimaging. (**d**) EndoV cleavage products for the IIUI-RNA substrates were run on 20% sequencing gels alongside with 10, 13 and 15 mer RNA markers. The illustration shows potential EndoV cleavage sites on the IIUI-RNA substrate. Abbreviations: - = no enzyme added, S = substrate, P = cleaved product, ss = single-stranded, ds = double-stranded, EcEV = *E. coli* EndoV. Enzyme amounts are shown in nM and sizes of RNA/DNA substrates and products are indicated in parentheses.

The mouse genome encodes a single EndoV homolog, mEndoV. The open reading frame of 1017 nucleotides translates into a protein of 338 amino acids with a calculated mass of 37.2 kDa. Mouse EndoV shares high sequence similarity with human EndoV except at the C-terminus which is also predicted to be structurally disordered and without evolutionary conservation^24^. Recent studies have revealed that hEndoV has a preference for inosines in RNA rather than DNA^17^,^21^ and we tested whether this was true for mEndoV as well. Indeed, when incubated with a single-stranded RNA substrate with a central inosine residue (I-RNA, Table 1), mEndoV was active, albeit cleavage was less efficient than for hEndoV (Fig. 1a,b). The double-stranded I-RNA substrate was a weaker substrate for mEndoV. Neither mEndoV nor hEndoV incised RNA without inosine, excluding these enzymes as general ribonucleases (Fig. 1b).

In cells multiple inosines are often found clustered, a phenomena referred to as RNA hyperediting^18^. To test such a substrate, a synthetic RNA with multiple inosines (IIUI-RNA) corresponding to part of exon 2 of rat α-tropomyosin gene was synthesized^25^. Mouse EndoV incised this substrate more efficiently both in a single- and double-stranded context (Fig. 1b). A site specific mEndoV mutant of the catalytic aspartate (D52A) totally lost the inosine-RNA incision activity for this as well as all other substrates tested (Fig. 1b).

Human EndoV has no activity on inosine-containing DNA substrates^17^ which applied to mEndoV as well (Fig. 1c). However, hEndoV efficiently cleaves a DNA substrate with inosine when a ribonucleotide is present immediately 3′ to the inosine residue^17^. This substrate (dIrG) was also incised by mEndoV confirming that both enzymes are critically dependent on a ribonucleotide for incision (Fig. 1b). Again the double-stranded substrate was less efficiently cleaved than the single-stranded version.

To map the exact cleavage position of the IIUI-RNA substrates, the reaction products were run on sequencing gels. The major incision products for both mEndoV and hEndoV corresponded to cleavage of the second phosphodiester bond 3′ of the second inosine (from the 5′ end) of the substrates (Fig. 1d). For the double-stranded substrate there was also some cleavage corresponding to the first inosine (from the 5′ end). For EcEndoV three cleavage products were seen, suggesting that all three inosines may accommodate in the nucleotide binding pocket. As the substrates were 5′-labelled, these assays will not reveal potential cleavage at the 3′ inosine prior to cleavage at the other inosines.

The catalytic properties of mouse and human EndoV were analyzed by testing different reaction conditions. Assays were performed at two pHs, 7.5 and 8.5, with different concentrations of Mn^2+^ and Mg^2+^ and with single- and double-stranded IIUI-and I-RNA substrates (Supplementary Fig. S1). The type and concentration of metal ion and pH did not change the activities of the two EndoVs notably, except that at pH 8.5, a Mn^2+^ concentration of 2.5 mM and higher inhibited activity. For further analysis two conditions were chosen; pH 8.5 with 2.5 mM Mg^2+^ (best activity) and pH 7.5 with 0.5 mM Mn^2+^ (close to physiological pH). The two buffers were used to test the effect of various Na^+^ and K^+^ concentrations on EndoV activity with the IIUI-RNA substrates. NaCl concentrations of 50, 100 or 150 mM did not change EndoV activity (Supplementary Fig. S2), except for double-stranded IIUI at pH 7.5 and 0.5 mM Mn^2+^ where inhibition was seen with increasing amounts of NaCl. Higher KCl concentrations than 50 mM (100 and 150 mM) inhibited both mouse and human EndoV activity (Supplementary Fig. S1). In sum, the two enzymes respond very similarly to changes in reaction conditions, and under all conditions tested, mouse EndoV is less active than human EndoV.

**Figure 2:**
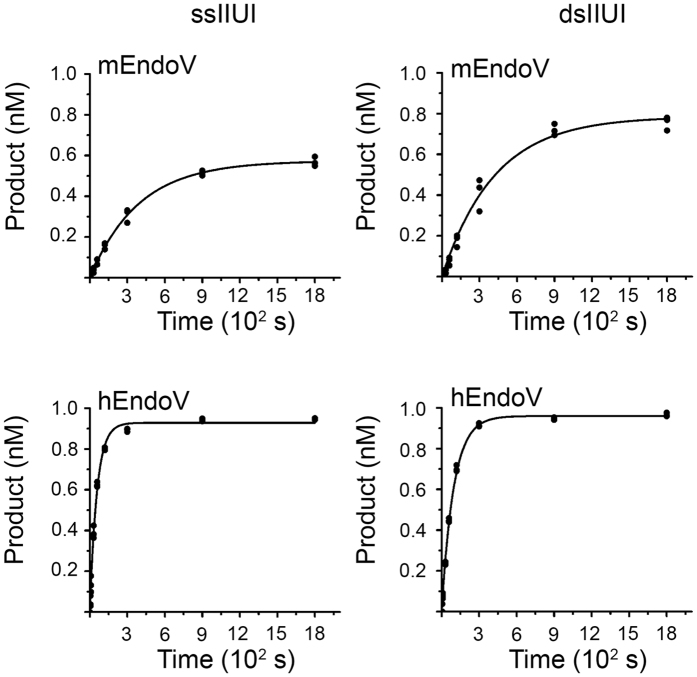
Single-turnover kinetics of mouse and human EndoV on ss and ds IIUI-RNA substrates. 30 nM enzymes were incubated with 1 nM ss IIUI-RNA or ds IIUI-RNA substrates and samples were withdrawn at time points as indicated and analyzed by PAGE. A one phase association model (solid line) was fitted to three parallel, independent experiments for calculation of the turnover rate *k*_obs_.

To compare the catalytic efficiencies of mEndoV and hEndoV, single-turnover kinetic analyzes were performed on the two IIUI-RNA substrates. The results revealed a 4–7 fold higher turnover rate for these substrates for hEndoV over mEndoV (Table 2, Fig. 2). Human EndoV had about a 2 times higher turnover rate for the single- versus double-stranded substrate (*k*_obs_, 0.0177 s^−1^ and 0.0104 s^−1^, respectively), a trend also previously shown for a single-inosine RNA substrate^17^, while mEndoV had the same turnover rate for cleavage of single- and double-stranded IIUI-RNA substrates (*k*_obs_, 0.0026 s^−1^ and 0.0024 s^−1^, respectively; Table 2).

**Figure 3:**
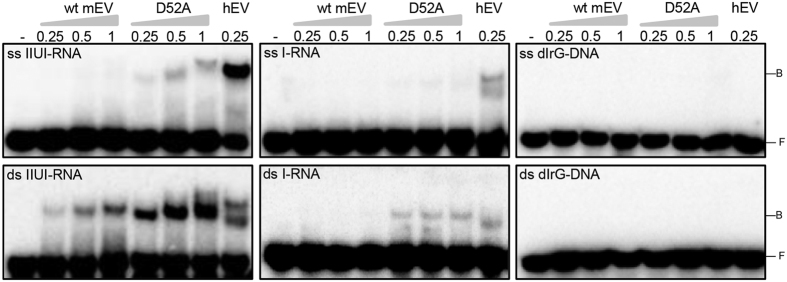
Binding properties of mEndoV to inosine containing substrates. Bandshift assays with wild type or mutant D52A mEndoV (0.25–1 μM) with ss/ds IIUI-RNA, ss/ds I-RNA and ss/ds dIrG-DNA substrates were performed. Human EndoV (0.25 μM) was included in all assays. After incubation on ice for 15 minutes, bound RNA/DNA (=B) were separated from free substrates (=F) by 10% native PAGE.

Human EndoV has been shown to interact and bind to different DNA and RNA substrates^21^,^24^. To test if mEndoV makes stable complexes with its substrates, electrophoretic mobility shift assays (EMSA) were done. Mouse EndoV barely made shifts with the single-stranded IIUI-RNA substrate, but bound stronger to the double-stranded IIUI-RNA substrate (Fig. 3). Under the conditions used, mEndoV bound much weaker than hEndoV, and no shift was observed for the I-RNA and dIrG-DNA substrates (Fig. 3). The catalytic inactive mEndoV mutant (D52A) made stronger shifts than the wild-type enzyme especially for the double stranded IIUI-RNA substrate, comparable in intensity to that of hEndoV. To verify that the bandshifts reflects substrate affinity and not product binding, the EMSA reaction products were analyzed by denaturing PAGE. Under the assay conditions used, no cleavage of the substrates was observed for none of the enzymes (data not shown).

### 3D structure of mEndoV

**Figure 4:**
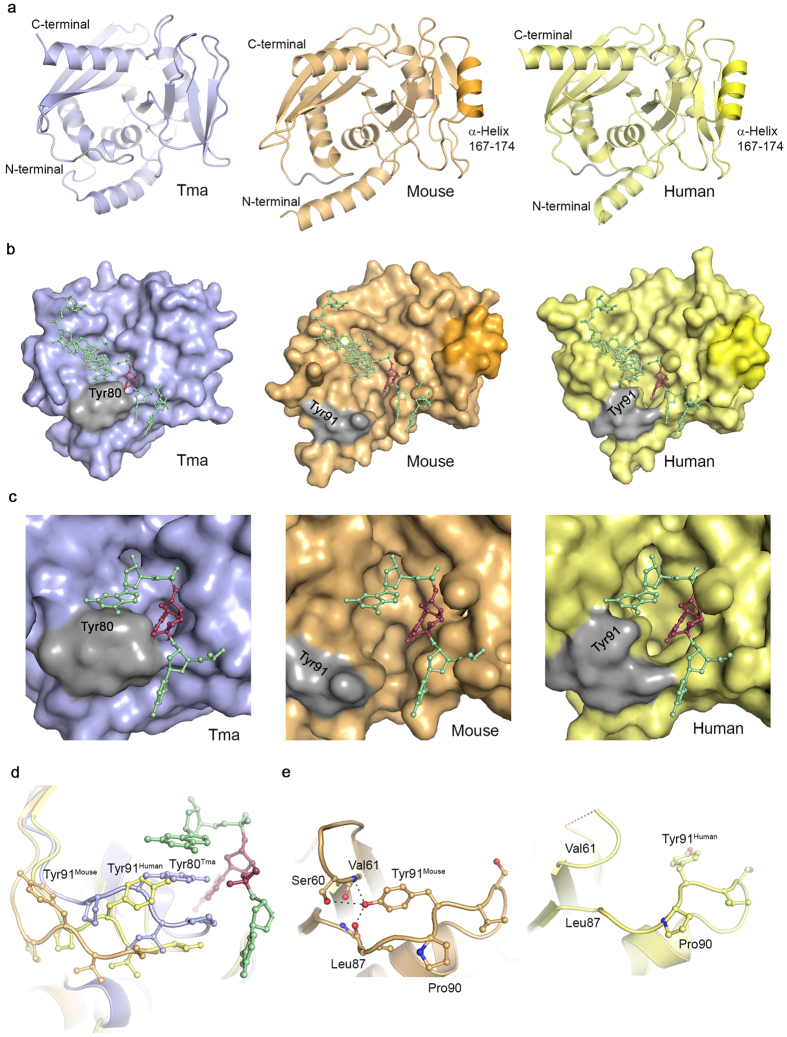
Endonuclease V structures. (**a**) Overall structure of *Thermotoga* (blue), mouse (orange) and human (yellow) EndoV. The mammalian versions used in crystallization have been truncated (mouse 1–250, human 13–250). The additional α-helix common to the mammalian variants is colored in a stronger hue in mouse and human EndoV. (**b**) Surface display of *Thermotoga*, mouse and human EndoV. The wedge residues are colored gray; the surface contour of the mammal-specific helix is shown for mouse and human EndoV in a stronger hue. The lesion-strand in DNA in the complex with TmEndoV has been overlayed onto the mouse and human EndoV structures. The inosine residue is colored dark red. (**c**) Close-up view of the binding pocket regions of *Thermotoga*, mouse and human EndoV, illustrating the open character of mouse and human EndoV compared with the more closed TmEndoV. (**d**) The conformation of the wedge motif in *Thermotoga* (blue), mouse (orange) and human EndoV (yellow). The TmEndoV structure is that of the DNA bound form with the inosine lesion in dark red. (**e**) Close-up view of the Tyr91 conformation in mouse and human EndoV. The hydrogen bond between mouse Tyr91, Ser60, Val61 and Leu87 are shown (left) and compared with the similar open region in hEndoV (right). The red dashed line in the loop in hEndoV indicates disordered residues not present in the structural model.

**Figure 5:**
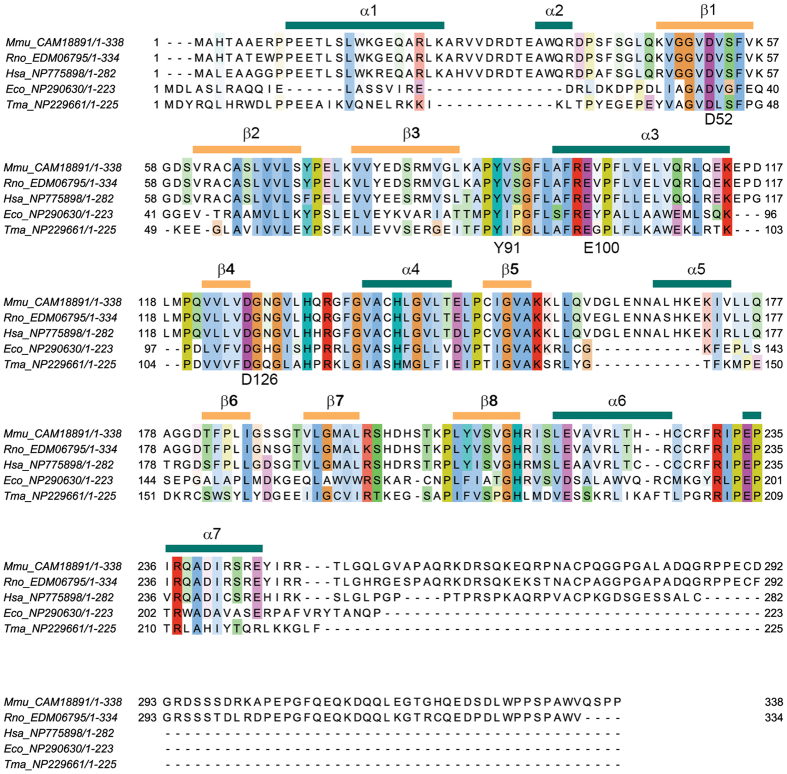
Multiple sequence alignment of EndoV from mouse (*Mmu*), rat (*Rno*), human (*Hsa*), *E*. *coli* (*Eco*) and *T*. *maritima* (*Tma*), including a secondary structure element assignment calculated from the mouse structure. Helices are denoted α1–α7 and extended β-strands β1–β8. The invariant Tyr91 and the three catalytic residues Asp52, Glu100 and Asp126 are labelled. The sequence reference codes for the NCBI protein database are listed. Sequences were aligned by Clustal Omega^41^ and the figure was made with the JalView software. The secondary structure element assignment was calculated with DSSP/WhatIf^42^,^43^.

The structure determination of mEndoV (Table 3) revealed a protein fold which is to a large extent similar to human and prokaryotic EndoVs (Fig. 4a)^12^,^26^. The first 9 residues on the N-terminal end are disordered with no specific conformation. The region around residues 167–174 contains a mammalian specific α-helix similar to that of hEndoV (Fig. 4a). The prokaryotic EndoVs are 11 residues shorter in this region, without formation of a helix (Fig. 5). A noticeable difference in the structure between the present mEndoV and previous reported EndoV structures is the conformation of the strand-separating wedge motif. This difference manifests itself in a quite open lesion binding pocket in mEndoV compared with Tm and hEndoV (Fig. 4b,c). The wedge motifs in hEndoV and also EcEndoV (PDB entry 4xpu)^27^ are retracted about 4–6 Å compared with the DNA bound TmEndoV, but the conserved Tyr91 in hEndoV^26^ is still pointing towards the DNA/RNA binding groove, with side chain torsion angle χ^1^ being ~−170° (*trans*) (Fig. 4d). Only a minor adjustment of the wedge segment is expected upon substrate binding. Contrary to this, in the structure of mEndoV, we find a different wedge conformation (Fig. 4d). The Tyr side chain points in the opposite direction, away from the DNA/RNA binding groove, with torsion angle χ^1^ being ~−40° (*gauche*^+^). The mEndoV is the first example of an EndoV structure with a “flipped” wedge. In contrast to all other reported EndoV structures, the invariant Tyr91 in mEndoV is interacting with the backbone of the protein through hydrogen bonds to Ser60, Val61 and Leu87 (Fig. 4e). In order to stack with nucleotides in the active site pocket, the mEndoV Tyr91 must undergo a transition to the *trans* side chain conformation for χ^1^. To look further into the difference in wedge conformation we performed a molecular dynamics (MD) simulation of both mouse and human EndoV.

### Molecular dynamics simulation of the wedge flexibility

The 100 nanoseconds simulation, with trajectory snapshots stored every 2.4 picosecond, reveal that Tyr91 in mEndoV moves between two side chain rotamers; χ^1^ ~−60° (*gauche*^+^) and χ^1^ ~ + 60° (*gauche*^−^), with a time distribution of about 3:1 (Supplementary Fig. S3). It never moves into the extended χ^1^ ~180° (*trans*) conformation that is found in the crystal structure of human EndoV. For human EndoV, the Tyr91 side chain moves between the crystal structure starting point χ^1^ ~180° (*trans*) and the χ^1^ ~−60° (*gauche*^+^), which corresponds to the conformation in the mouse EndoV structure. The side chain oscillates between the two conformations with a time distribution of about 1:1. These simulations support our structural data showing that the wedge motif has some plasticity and that transitions between different conformations can take place.

## Discussion

A series of prokaryotic and eukaryotic EndoV homologs have recently been studied with respect to catalysis, substrate specificity and structure^12^,^21^,^26^,^27^,^28^. The human homolog has activity for inosines in RNA, while the bacterial versions can cleave both RNA and DNA with inosines. Here we have characterized the activity profile of EndoV from mouse and present its 3D structure. Except for the structure of hEndoV, only bacterial EndoV enzymes have been structurally solved to date. Our data show that mEndoV shares substrate preferences with hEndoV by catalyzing cleavage at inosines in RNA and being inert towards inosines in DNA. Mouse EndoV seems to prefer RNA substrates with clustered inosines, however the turnover rate for the best substrate tested, ss IIUI-RNA, is about 7-fold lower than that of hEndoV.

Both the activity and the bandshift analyzes show that mEndoV favours substrates with multiple inosines. Recent publications of RNA sequencing data sets have revealed that also in the mouse transcriptome, the vast majority of A-to-I editing sites are found outside coding exons in intronic or untranslated regions consisting of inverted repeats^29^,^30^. It might be that mEndoV activity is adapted to hyperedited RNA rather than site-selective deamination sites. Under the reaction conditions used, both cleavage and substrate binding by mEndoV appeared less efficient than for hEndoV. This could be related to the relatively wide RNA/DNA binding groove and the orientation of the tyrosin (Tyr91) away from the active site as revealed by the mEndoV structure. In the TmEndoV structure this tyrosin is involved in base stacking with DNA, contributing to stabilisation of the enzyme-substrate complex. The much wider RNA binding cleft of mEndoV seems to allow more flexibility in the protein, possibly also with different substrate binding modes than TmEndoV. From the sequencing gel it appears that the second (middle) inosine in the hyperedited 5′…IIUI…3′ substrate is preferred, probably by making more stable interactions with the enzyme using a binding conformation that positions the second inosine in the nucleotide binding pocket. In line with this, the strongest shift in EMSAs for mEndoV was found with this substrate.

The structure of mouse EndoV reveals a new conformation of the highly conserved strand-separating wedge motif. The invariant Tyr residue, normally pointing into the DNA/RNA binding groove and stacking with the DNA/RNA substrate during catalysis, is facing away from the groove. For this reason, the DNA/RNA binding groove in the crystal structure of mouse EndoV in the unbound state is wide and shallow. The MD simulation suggests that the wedge motif has some flexibility and that the invariant Tyr might flip between two conformations, of which a probably unbound or unproductive state is shown in the structure of mEndoV. We cannot exclude that the observed difference in Tyr conformation and wedge backbone trace might be due to crystal packing effects, however, the MD simulation suggests that the wedge motif is more flexible than foreseen from previous structures, and that the present mouse EndoV structure has captured an ‘open’ state of the wedge.

The structure also unveils a surface-exposed α-helix not present in the prokaryotic forms of EndoV but also found in the human homolog^26^. This element is on the outskirts of the DNA/RNA binding cleft, and may serve a regulatory or coordinative role in protein-protein interactions. Actually, an observed “head-to-shoulder” dimerization in the structure of EcEndoV is suggested to affect catalytic performance, as shown by reduced activity for the EcEndoV Ser144Ala single mutant and the Glu140Arg/Ser144Ala double mutant^27^. Intriguingly, the α-helix unique to the mammalian variants corresponds to the proposed dimerization region in EcEndoV. In hEndoV, the quadruple-cysteine motif 225-CCCC-228 was shown to be important for catalysis, with serine mutations of Cys227 and Cys228 reducing the activity by 68 and 46%, respectively^26^. The molecular basis for the influence of these two cysteine residues on hEndoV catalysis is not known. The corresponding region in mEndoV is 225-HHCC-228, and our structure of mEndoV reveals that the first two Cys residues in hEndoV are replaced by histidines with no change in the local geometry. The latter two Cys residues are conserved between mouse and human EndoV, and with similar conformations and local environment in the two structures. Our present structure does not suggest a molecular explanation for the importance of these residues in catalysis, but it is tempting to suggest that the effect is due to protein stability in general, since EcEndoV and TmEndoV have strong activities even with Cys/Met and Phe/Thr in these positions, respectively.

In this study, we have shown that mouse EndoV is active on RNA substrates containing one or several inosines. In particular, our data revealed that hyperedited substrates, such as IIUI-RNA, are favored. The biological significance of the partition of activity between mammalian and prokaryotic EndoVs, operating on only RNA or both DNA and RNA respectively, is still to be investigated. The active site in mouse and human EndoV are rather similar to EcEndoV and TmEndoV, hence the difference in activity probably reside in substrate recognition (RNA versus DNA) or the specific need for a 2′ hydroxyl group in the nucleotide 3′ to the inosine aligned in the nucleotide binding pocket. Structures of EndoV in complex with RNA substrates will reveal detailed information on the molecular basis of RNA recognition and incision by mammalian EndoV.

## Methods

### Purification of mEndoV

Codon optimized mouse EndoV cDNA (NP_001164636.1) was cloned into pETM41 (EMBL, Heidelberg) in frame with the N-terminal His-maltose binding protein (His/MBP) tag (Genescript). Mouse EndoV proteins were expressed in a modified version of *E. coli* ER 2566 (ER2566 Δ*nfi* Δ*rnhB*) where the endogenous *nfi* and *rnhB* genes had been inactivated by transformation with the kanamycin-deletion cassettes derived from the JW5547-1 (*nfi::kan*) and JW0178-1 (*rnhB::kan*) strains purchased from the Keio collection^31^. Correct integrations and subsequent excision of the cassettes by the use of the Flp recombinase encoded by pCP20 plasmid^32^, were verified by colony PCR. Cells were grown in Lysogeny broth (LB) medium, supplemented with 50 mg/l kanamycin at 37 °C until OD_600_ reached 0.6–0.7. The temperature was lowered to 18 °C, protein expression induced with 1 mM IPTG and cells incubated at 18 °C over night. Cells were harvested by centrifugation, and the cell pellet dissolved in amylose buffer (50 mM Tris pH 8.0, 200 mM NaCl, 1 mM EDTA, 5% glycerol and 10 mM β-mercaptoethanol (β-ME)). Cells were sonicated (3 × 30 s) followed by centrifugation (27 000 g, 30 min). The supernatant was incubated with amylose resin with gentle shaking at 4 °C and after 1 h transferred to a column. Fusion proteins were eluted with maltose-buffer (50 mM Tris pH 8.0, 200 mM NaCl, 1 mM EDTA, 10 mM β-ME, 5% glycerol, 10 mM maltose). Fractions were analyzed by SDS-polyacrylamide gel electrophoresis (PAGE) and peak fractions pooled. After concentration (Amicon ultra-4, MWCO 10 kDa), proteins were applied to a Superdex 75 size-exclusion column using buffer 50 mM Tris pH 8.0, 50 mM NaCl, 10 mM β-ME. Fractions rich in EndoV were pooled and subjected to TEV protease cleavage (1:100 ratio in mg) by dialysis in 50 mM Tris pH 8.0, 1 mM DTT, 0.5 mM EDTA, 5% glycerol at 4 °C over night. To remove the released His-MBP tag, the protein solution was directly applied to a HiTrap Q ion exchange chromatography column. Free mEndoV did not bind to the HiTrap Q column and the flow through was then applied to a Resource S column after adjusting the final concentration of NaCl and MES pH 6.0 to 100 mM. Mouse EndoV was eluted in the flow through and stored at −80 °C in 20% glycerol. hEndoV was purified as previously described (ref 17).

For crystallisation, a site specific (D52A) truncated (residues 1–250) mutant of mEndoV fused to His/MBP in pETM41 was expressed in BL21 CodonPlus(DE3)-RIPL *E. coli* cells. The cells were grown in LB medium at 37 °C, and protein production was induced by the addition of 0.5 mM IPTG at OD_600_ around 0.5–0.6 and incubation continued at 37 °C for 2 hours. Cells were harvested and the cell pellet was dissolved in cold buffer containing 50 mM Tris pH 8.0, 300 mM NaCl, 10 mM imidazole and 10 mM β-ME (buffer A). A cell-free extract was prepared by sonication and centrifugation. The extract was applied to a Ni-NTA agarose column and after washing extensively with buffer A, the His/MBP-mEndoV fusion protein was eluted using 50 and 300 mM imidazole in buffer A. The fractions rich in His/MBP-mEndoV were pooled, concentrated and supplemented with TEV protease (1:100 ratio) and dialyzed at 4 °C for 72 h against a buffer with 50 mM Tris pH 8.0, 10 mM β-ME and 0.5 mM EDTA. The protein solution was further dialysed against buffer A, and the free mEndoV was separated from TEV, His/MBP and uncleaved His/MBP-mEndoV by a second round of purification using Ni-NTA. The flow-through and wash fractions rich in mEndoV were concentrated as above and dialyzed to lower the pH in two stages using 50 mM MES pH 6.5, 100 mM NaCl and 10 mM β-ME, followed by 50 mM MES pH 6.0, 100 mM NaCl, and 10 mM β-ME. The protein was then applied to a Resource S column and eluted using a gradient in NaCl from 50 mM to 2M with 50 mM MES pH 6.0 and 10 mM β-ME. The pure mEndoV protein was concentrated to ~13 mg/ml for crystal screening.

### Cleavage and electrophoretic mobility shift assays

Oligonucleotide substrates were from Eurofins (DNA) and Midland Certified (RNA) (Table 1). The oligonucleotides were end-labelled using T4 polynucleotide kinase (New England Biolabs) and [γ^32^P] ATP (3000 Ci/mmol, Amersham). Double stranded substrates were generated by annealing the labelled oligonucleotide to a complementary strand. 10 μl reaction mixture contained 1 nM substrate, amount of enzymes as indicated and standard reaction buffer (10 mM Tris-HCl pH 7.5, 0.5 mM MnCl_2_, 50 mM KCl, 1 mM DTT and 5% glycerol), unless otherwise stated. Reactions proceeded at 37 °C for 30 min and stopped by adding 10 μl formamide loading dye (80% formamide, 10 mM EDTA, 0.1% xylene cyanol and bromphenol blue). The oligonucleotides were denaturated at 52 °C for 2 min, and the reaction products separated on 20% polyacrylamide/urea gels at 200 V for 1 h in 1x taurine buffer. The radiolabeled fragments were visualized by phosphorimaging (Typhoon 9410 Variable Mode Imager) and ImageQuant TL was used for quantification. All assays were performed 2–3 times and representative experiments are shown. Denaturing sequencing gels (20%) were run in 1x taurine at 35 W for 1 h.

For the single turnover assays, we first titrated 3–100 nM of mouse EndoV and the experiment showed that above ~25 nM enzyme concentration, we satisfy single-turnover conditions (data not shown). Thus, 30 nM EndoV was incubated with 1 nM substrate using the same reaction buffer as above. Samples were withdrawn as indicated in the figure and reactions stopped and analyzed as above. Samples were stored on ice until all time points were finished. For the calculation of the catalytic turnover rate *k*_obs_ (s^−1^), a one phase association model was fitted to three parallel data sets.

In electrophoretic mobility shift assays, enzymes (0.25–1 μM) and substrates (1 nM) were mixed with band shift buffer (40 mM Tris-HCl pH 8.5, 10 mM CaCl_2_, 10 mM DTT, 6% glycerol) and tRNA (1 ng/ μl) as a competitor. Samples were incubated on ice for 15 min, added DNA loading buffer (Thermo Scientific) prior to separation on 10% native polyacrylamide gels in 1x taurine with 5 mM CaCl_2_ at 100 V for 40 min at room temperature. The enzyme-substrate complexes were visualized as above.

### Crystallization, diffraction data collection, structure determination and model refinement

The D52A mutant of mEndoV was screened for crystallization using a Mosquito robot (TTP Labtech) and several commercial crystallization kits. Crystals appeared in condition 48 of the JCSG + kit, which contains 16% PEG 8000, 40 μM KH_2_PO_4_ and 20% by volume of glycerol. Crystals were transferred to a cryogenic solution made by mixing the reservoir solution with 10% ethylene glycol. After a short soak (<5 s) the crystals were flash-frozen in liquid nitrogen. X-ray diffraction images were collected at the ID23-2 beamline at the European Synchrotron Radiation Facility (ESRF). Data was collected at T = 100K using a wavelength of λ = 0.8729 Å. The data was integrated, scaled and analyzed using MOSFLM^33^, Aimless^34^ and CCP4i^35^. The structure was solved by molecular replacement using a poly-alanine version of the structure of *Thermotoga maritima* EndoV (PDB entry 4B20)^36^, as an input search template to Phaser^37^. The model was autobuilt into the mouse EndoV sequence using PHENIX^38^. The final refinement and model adjustments were done using PHENIX and Coot^39^. A few side chains have alternate positions, and these were modelled with 50% occupancy for each conformation. 8 residues in the N-terminus and 3 residues in the C-terminus are not visible in the electron density map, and are not included in the final model, and a few more residues at the C-terminal end have been modelled with 50% occupancy. Final atomic coordinates and structure factors have been deposited in the Protein Data Bank with accession code 5aoy.

### Molecular dynamics simulation

The preparation and simulations of mouse and human EndoV were carried out using the Desmond molecular dynamics (MD) program (version 4.4)^39^ within the Maestro modelling software GUI from Schrödinger (release 2015-4). The crystal structures of mouse and human EndoV without solvent water molecules served as starting points for the MD simulation. For human EndoV, the missing residues Lys57 and Gly58 were filled in using Coot^40^ and the loop was given an idealized geometry using the ‘Regularize zone’ tool of Coot. Each structure was pre-processed and energy minimized using the ‘Protein preparation’ tool in Schrödinger. Hydrogen atoms were added, the N- and C-terminal residues were capped and the hydrogen bond network optimized before energy minimization. The minimized protein structures were enclosed by solvated boxes filled with water molecules in addition to Na^+^ and Cl^−^ ions corresponding to a 150 mM buffer. The systems were again minimized and relaxed by short MD simulations using the default ‘Relax model system’ protocol in Desmond before starting 100 nanoseconds simulations with periodic boundary conditions. The simulation temperature was set to 300K, and both temperature and pressure were kept constant during the simulation (NTP ensemble simulation). Coordinates were stored every 2.4 picoseconds, forming simulation trajectories with more than 41000 structural snapshots. An inspection of the all-C_α_ RMDS value for all structural snapshots relative to the starting point structure shows that both systems seems to equilibrate within the first 10–15 nanoseconds, with RMSD values fluctuating between 1.6–1.8 and 1.8–2.0 Å for mouse and human EndoV, respectively. There was a short period around 70 ns where the RMSD of human EndoV increased to ~2.8 Å over a 10 ns period, but the system subsequently returned to baseline. The trajectories were used to extract time-laps profiles of the side chain rotamers of Tyr91. The simulations were run on the Stallo computing cluster at the University of Tromsø, Norway, using 64 CPU-cores for each simulation.

## Additional Information

**How to cite this article**: Nawaz, M. S. *et al.* Crystal structure and MD simulation of mouse EndoV reveal wedge motif plasticity in this inosine-specific endonuclease. *Sci. Rep.* **6**, 24979; doi: 10.1038/srep24979 (2016).

## Supplementary Material

Supplementary Information

## Figures and Tables

**Table 1:** Oligonucleotide substrates used in the study.

5′ → 3′ sequence	DNA/RNA	Description
CCGUAGAGCUAC[rI]GAUCGGUCACCG	RNA	I-RNA
CGGUGACCGAUCUGUAGCUCUACGG	RNA	Complementary oligo for I-RNA
ACUGGACAAAUACUCCGAGG	RNA	ctr-RNA
CCUCGGAGUAUUUGUCCAGU	RNA	Complementary oligo for ctr-RNA
ACUGGACA[rI][rI]U[rI]CUCCGAGG	RNA	IIUI-RNA
CCUCGGAGU[rI]UUUGUCCAGU	RNA	Complementary oligo for IIUI-RNA
CCGTAGAGCTAC[dI][rG]ATCGGTCACCG	DNA	dIrG-DNA
CCGTAGAGCTAC[dI]GATCGGTCACCG	DNA	I-DNA
CGGTGACCGATCTGTAGCTCTACGG	DNA	Complementary oligo for dIrG/I-DNA

**Table 2:** Single-turnover kinetics rates (*k*_obs_) with standard deviations for mouse and human EndoV on ss IIUI-RNA and ds IIUI-RNA substrates.

Enzyme	Substrate	*k*_obs_ (s^−1^)
Human EndoV	ss IIUI-RNA	0.0177 ± 0.0006
	ds IIUI-RNA	0.0104 ± 0.0003
Mouse EndoV	ss IIUI-RNA	0.0026 ± 0.0001
	ds IIUI-RNA	0.0024 ± 0.0002

Standard-deviations are calculated from curve fitting to three independent data sets.

**Table 3:** Crystal data, data-collection statistics and refinement data.

Data Collection	
Beamline	ID23-2 (ESRF, Grenoble)
Wavelength (Å)	0.8729
Space Group	*C*222_1_
Unit-cell parameters (Å, °)	*a* = 101.63, *b = *114.03, *c = *54.01
	*α = β = γ = *90
Resolution (Å)	32.47-1.75 (1.78-1.75)^a^
Unique reflections	32035 (1744)
Completeness (%)	99.9 (99.7)
Multiplicity	4.0 (3.9)
Mean I/σI	11.0 (3.0)
R_meas_ (all I^+^ and I^−^)^b^	0.098 (0.537)
Refinement statistics	
Resolution of data used in refinement (Å)	32.47–1.75
Reflections used in refinement	31483
Completeness (%)	98.2
R_cryst_/R_free_ (%)^c^	16.5/19.0
R.m.s.d. bonds (Å)	0.007
R.m.s.d. angles (°)	1.19
Average B-factor (protein/solvent) (Å^2^)	24.3/38.4
Number of atoms	
Protein	1896
Solvent	302
Ramachandran plot (%)^d^	
Favorable region	98.4
Additionally allowed	1.2
Outliers	0.4

^a^Values in parentheses are for the highest resolution shells.

^b^R_meas_ defined by Diederichs & Karplus^44^.

^c^R_cryst_ = Σhkl ||*F*_O_|-|*F*_C_||/Σhkl |*F*_O_| where *F*_*O*_ and *F*_C_ are the observed and calculated structure factor amplitudes, respectively. R_free_ is calculated from a randomly chosen 5.08% set of unique reflections not used in refinement.

^d^Defined using MolProbity^45^.
