# Supplementary material for: Crystal structure and MD simulation of mouse EndoV reveal wedge motif plasticity in this inosine-specific endonuclease
Source: Sci Rep. 2016 Apr 25;6:24979. doi: 10.1038/srep24979 (PMC4842958; doi:10.1038/srep24979)
Supplement: Supplementary Information [file srep24979-s1.pdf]

Crystal structure and MD simulation of mouse EndoV reveal wedge motif plasticity in this inosine-specific endonuclease

Meh Sameen Nawaz, Erik Sebastian Vik, Mia Elise Ronander, Anne Marthe Solvoll, Pernille Blicher, Magnar Bjørås, Ingrun Alseth, Bjørn Dalhus

Supplementary Information

Supplementary Figure S1

Influence of pH and cofactor concentrations on EndoV activity. Mouse (grey curves) and human (black curves) endonuclease V were incubated at pH 7.5 (square) or pH 8.5 (circle) with increasing amounts of $MnCl_2$ (left panels) or $MgCl_2$ (0.5-2.5-5 mM) (right panels) and with substrates (**a**), (**b**); single stranded IIUI, (**c**), (**d**); double stranded IIUI, (**e**), (**f**); single stranded I, (**g**),(**h**); double stranded I. In **a-d** 15 nM enzymes were used, and in **e-h** 25 nM. Assays were performed as described for Figure 1b. EndoV activity without Mn/Mg ions was set to 0% in all assays. Cleavage (%) was quantified using ImageQuant TL.

Supplementary Figure S2

Influence of Na^+ and K^+ concentrations on Endonuclease V activity. Mouse (grey curves) and human (black curves) endonuclease V were incubated with increasing amounts of KCl (square) or NaCl (circle) (50-100-150 mM) at pH 7.5, 0.5 mM MnCl2 (left panels) or at pH 8.5, 2.5 mM MgCl2 (right panels) and with substrates (**a**), (**b**); single stranded IIUI, (**c**), (**d**); double stranded IIUI. 15 nM enzymes were used and assays performed as described for Figure 1b. ImageQuant TL was used for quantification.

Supplementary Figure S3

Tyr91 side chain conformation in MD simulations of mouse and human EndoV. (**a**) In mouse EndoV, the Tyr91 χ^1 side chain fluctuates between conformation ~ -60° and ~ +60°, with time ratio ~ 3:1. (**b**) In human EndoV, the Tyr91 χ^1 side chain fluctuates between conformation ~ +/-180° and ~ -60°, with time ratio ~ 1:1.

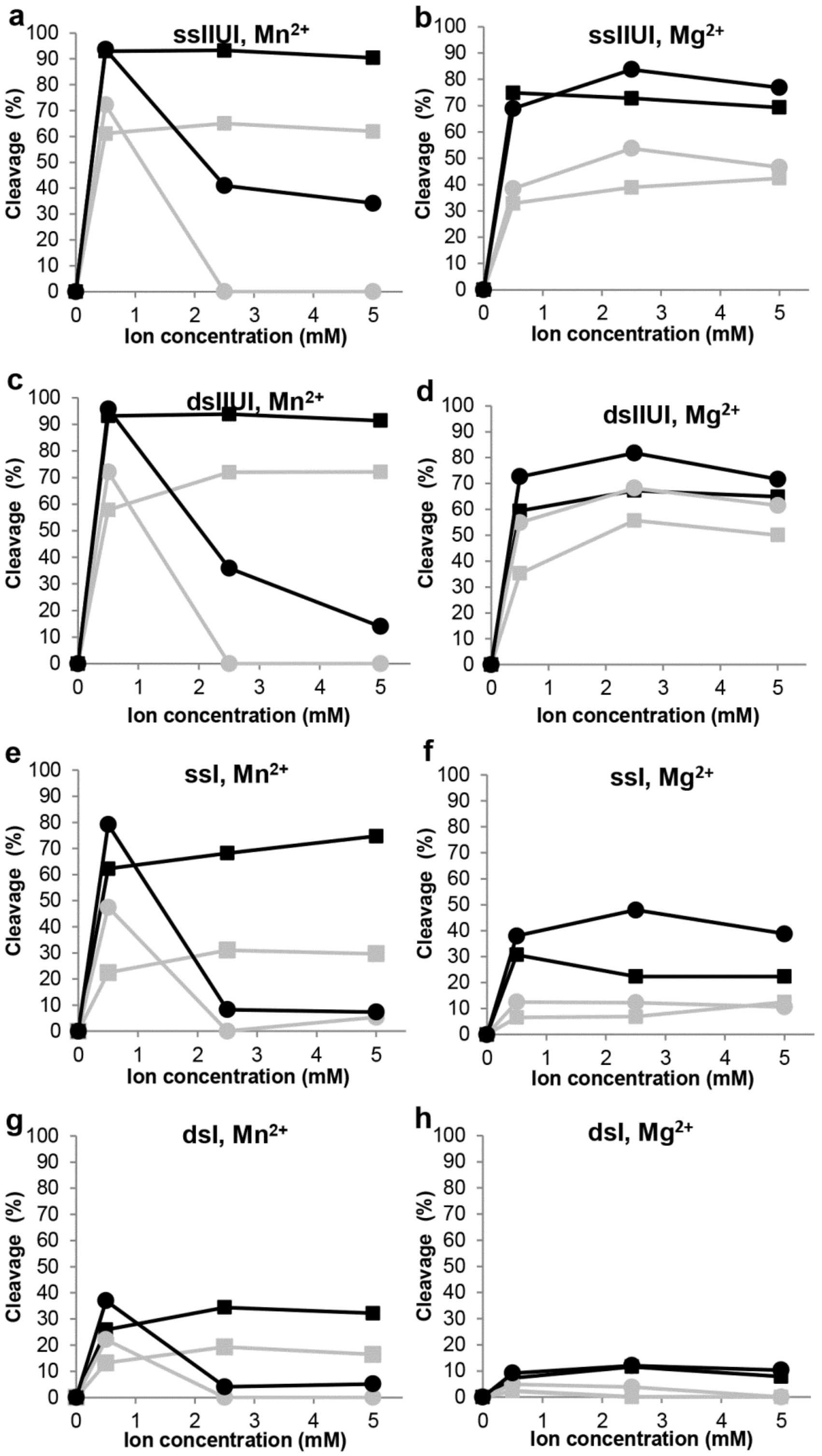

Supplementary Figure S1

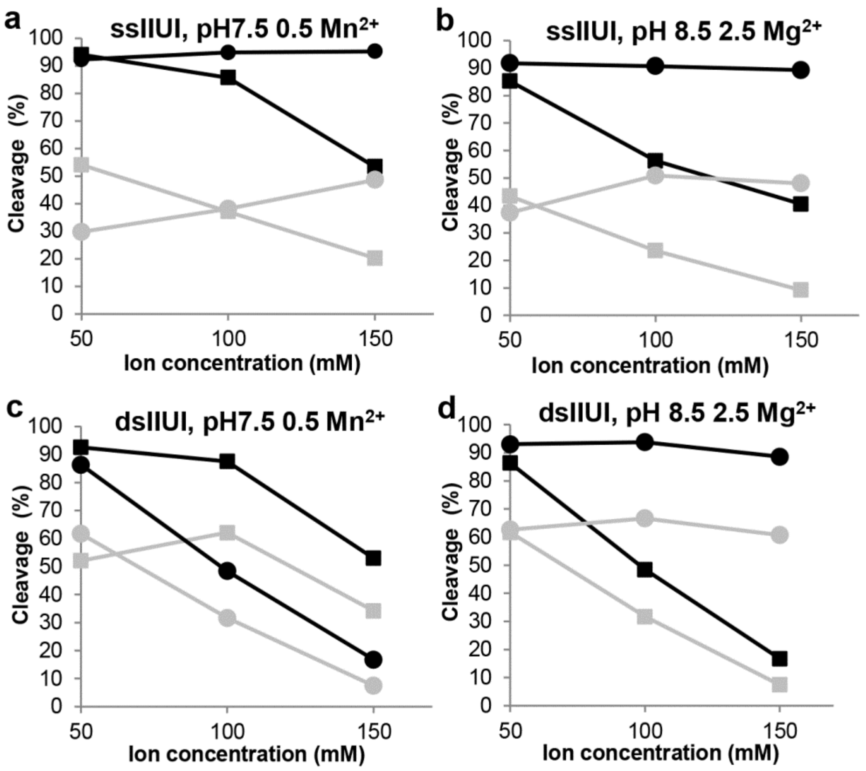

Supplementary Figure S2

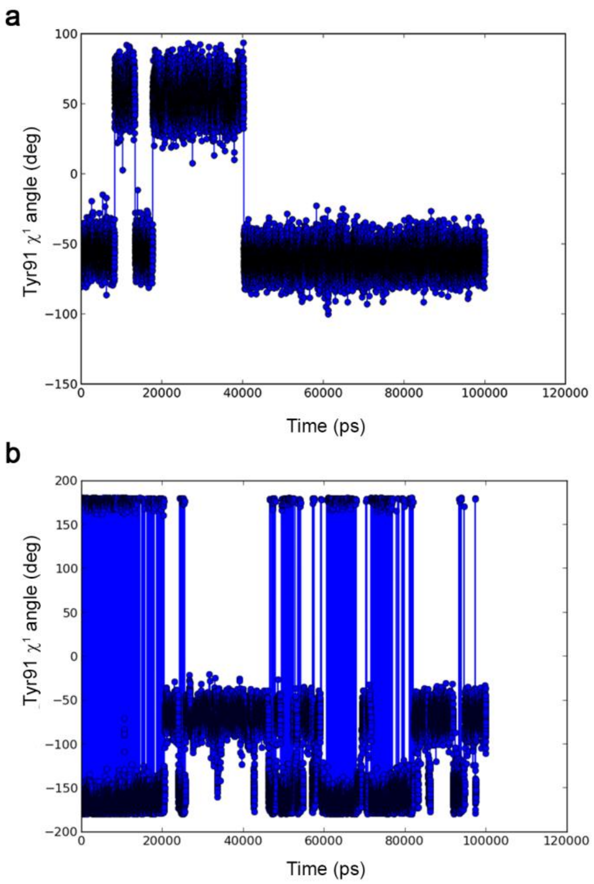

Supplementary Figure S3
